# Supplementary material for: Single-cell, whole-embryo phenotyping of mammalian developmental disorders
Source: Nature. 2023 Nov 15;623(7988):772–81. doi: 10.1038/s41586-023-06548-w (PMC10665194; doi:10.1038/s41586-023-06548-w)
Supplement: Supplementary file 1 — Supplementary Note 1 and legends for Supplementary Tables 1–7. [file 41586_2023_6548_MOESM1_ESM.docx]

**Supplementary Notes**

Supplementary Note 1: Potentially incorrect staging of *Scn11a* GOF mutants

The *Scn11a* GOF mutant exhibited the most extreme similarity scores, in terms of both similarity between replicates and dissimilarity with other genotypes (**Fig. 4a**; **Extended Data Fig. 10a**). The *Scn11a* GOF mutant carries a missense mutation in the *Scn11a* locus which is reported to result in reduced pain sensitivity both in mice and men without obvious signs of neurodegeneration, suggesting altered electrical activity of peripheral pain-sensing neurons and impaired synaptic transmission to postsynaptic neurons[^21^](https://paperpile.com/c/UCFxsq/zD3vo). However, at least grossly, the mutant does not seem to be associated with mesenchymal phenotypes. Noting that the *Scn11a* GOF mutant embryos clustered with E12.5 embryos instead of E13.5 embryos in our pseudobulk analysis (**Fig. 1c**), we speculated that its extreme similarity scores might be attributable either to developmental delay of the *Scn11a* GOF mutant at the scale of the whole embryo or incorrect staging. To investigate this further, we co-embedded *Scn11a* GOF mutant cells with pooled wildtype cells and MOCA cells from the neural tube trajectory. While wildtype cells were distributed near E13.5 cells from MOCA, the *Scn11a* GOF cells were embedded closer to cells from earlier developmental timepoints (**Extended Data Fig. 10d**). As a more systematic approach, we calculated a “time score” for each cell from the MMCA dataset by taking the k-NNs of each MMCA cell in the MOCA dataset and calculating the average of the developmental time of the MOCA cells. The relative time score distributions of *Scn11a* GOF cells and wildtype cells suggest that *Scn11a* GOF cells are significantly delayed in all major trajectories examined (single sided student’s t-test, raw p-value < 0.01; **Extended Data Fig. 10e**). To further follow up on the possibility of earlier time points being inadvertently harvested for this mutant, we examined 2 mixed litters of wildtype and heterozygous mutants at the stage E13.5 (**Extended Data Fig. 10g**). As the heterozygous mutants did not exhibit signs of developmental delay such as smaller size, difference in eye formation or limb development, the theory of general developmental delay seems unlikely, as a more parsimonious explanation is incorrect staging of these litters.

**Supplementary Tables**

Supplementary Table 1. Brief descriptions of individual mutant types

Supplementary Table 2. Marker genes used for mouse developmental trajectory annotation.

Supplementary Table 3. Cell composition changes for individual mutants across developmental trajectories.

Supplementary Table 4. Marker genes used for annotating the subtypes of selected cell populations.

Supplementary Table 5. lochNESS related genes identified with regression model in Gli2 KO and pooled wildtype in the roof plate sub-trajectory

Supplementary Table 6. Quantification of Ttr stainings

Supplementary Table 7. lochNESS related genes identified with regression model in Tbx3 and pooled wildtype in selected epithelial sub-trajectories
